# Depression and antidepressant treatment in growing up in New Zealand: impact on preterm birth and low birth weight

**DOI:** 10.1007/s00737-026-01692-4

**Published:** 2026-03-21

**Authors:** Stephanie D’Souza, Francesca Anns, Karen E. Waldie, Roger T. Mulder, Julia J. Rucklidge

**Affiliations:** 1 Centre of Methods and Policy Application in the Social Sciences, Faculty of Arts and Education, University of Auckland, Auckland, New Zealand; 2 School of Social Sciences, Faculty of Arts and Education, University of Auckland, Auckland, New Zealand; 3 School of Psychology, Faculty of Science, University of Auckland, Auckland, New Zealand; 4 Department of Psychological Medicine, University of Otago, Dunedin, New Zealand; 5 School of Psychology, Speech and Hearing, University of Canterbury, Christchurch, New Zealand

**Keywords:** Antenatal depression, Antidepressant use, Maternal mental health, Preterm birth, Low birth weight, Gestational age, Birth outcomes

## Abstract

**Purpose:**

To investigate the independent effects of antenatal depression and antidepressant use on birth weight and gestational age in a large, ethnically diverse New Zealand cohort.

**Methods:**

Data were obtained from 6,759 pregnancies in the *Growing Up in New Zealand* longitudinal study, of which 5,200 (76.9%) involved neither antidepressant exposure nor unmedicated depression, 197 (2.9%) involved antidepressant exposure, and 715 (10.6%) involved unmedicated depression. Antenatal depression was assessed using the Edinburgh Postnatal Depression Scale (EPDS), and prenatal antidepressant use was self-reported. Birth outcomes were obtained through linked perinatal health records. Multiple regression analyses examined continuous and categorical outcome definitions, adjusting for sociodemographic and antenatal health variables.

**Results:**

The prevalence of antidepressant use and unmedicated antenatal depression (EDPS *≥* 13) was 2.9% and 10.6%, respectively. Antidepressant use predicted a 0.40-week reduction in gestational age (95% CI: -0.55 to -0.26), and each one-point increase in EPDS score predicted a 0.02-week reduction (95% CI: -0.03 to -0.01). Higher EPDS scores were also associated with increased odds of preterm birth (OR = 1.04, 95% CI: 1.02 to 1.07) and low birth weight (OR = 1.03, 95% CI: 1.00 to 1.06).

**Conclusions:**

Antidepressant use and antenatal depression were each associated with slightly earlier births, but only depressive symptoms were associated with increased risk of preterm birth and low birth weight. Effect sizes were small. Antidepressants remain an important, and often most accessible treatment option, for maternal depression. Greater investment in screening and culturally responsive, non-pharmacological options is also needed to support diverse preferences and wellbeing.

## Introduction

Antenatal depression affects between 15 and 65% of pregnant women worldwide (Dadi et al. 2020; Yin et al. 2021). In New Zealand (NZ), 11.9% of women are screened as likely experiencing depression in their third trimester, indicated by an Edinburgh Postnatal Depression Scale (EPDS) score of ≥ 13 (Waldie et al. 2015). Rates are disproportionately higher among Māori, Pacific and Asian women compared to those of European descent. Although NZ lacks prevalence estimates derived from diagnostic tools, the pooled prevalence of perinatal depression diagnoses in high income countries is 9.3% (Woody et al. 2017).

Untreated antenatal depression is linked to higher rates of adverse birth outcomes and complications, such as preterm birth (PTB), low birth weight (LBW), increased neonatal intensive care use and 5-min Apgar scores, and a greater need for medical interventions (Benatar et al. 2020; Jahan et al. 2021; Jarde et al. 2016; Khanghah et al. 2020; Simonovich et al. 2021; Sun et al. 2021). An umbrella review identified that infants born to people experiencing depression are 1.49 times more likely to be LBW and 1.40 times more likely to be PTB (Dadi et al. 2020). These outcomes can negatively affect early development, with lasting physical and mental health impacts into adulthood (Anderson et al. 2021; Crump 2020). PTB and LBW complications are the leading cause of death in the first five years (Ream and Lehwald 2018).

Clinical guidelines for antenatal depression largely align with those for other life stages, with additional considerations for pregnancy and foetal development (Molenaar et al. 2018). For moderate to severe or treatment-resistant depression symptoms, antidepressants alongside psychological interventions are recommended (Austin et al. 2017; Malhi et al. 2021). In NZ, estimates indicate that 3.2%−6.6% of pregnant women take antidepressants (Broughton et al. 2023; Svardal et al. 2021), with evidence of increasing trends over time (Donald et al. 2021). However, access to psychological treatments remains limited, with barriers existing across multiple levels of care (Sambrook Smith et al. 2019).

Given the lack of controlled studies on prenatal antidepressant use, observational studies guide clinical decision-making. These studies can help inform discussion with expectant mothers on the risks and benefits of antidepressant use in pregnancy. However, distinguishing what is associated with antidepressants from what is related to other confounding variables, including underlying mental health conditions and their severity, is challenging.

Other maternal characteristics linked to adverse birth outcome risk include maternal age, ethnicity, smoking history, socio-economic status, low educational achievement, obstetric history and pregnancy planning, substance use, nutritional status, and diet quality (Englund-Ögge et al. 2019; Fuchs et al., 2018). Multiple health risks further increase the likelihood of adverse health outcomes for both mother and child (Bird et al. 2017). These risk factors can disproportionately cluster among those with depression or on antidepressants, complicating interpretations.

Consequently, observational studies and meta-analyses show considerable variability in risks associated with antidepressants. Identified associations of in-utero antidepressant exposure include PTB, LBW and higher admission rates to specialised care (Bandoli et al. 2020; Hogue et al. 2017; Huang et al. 2014; Ulbrich et al. 2021). Other studies find that risks are eliminated once confounding effects are controlled for, or not considered clinically significant as outcomes of exposed women are typically within normal ranges (Amit et al. 2024; Ross et al. 2013). Nevertheless, pregnant women are often reluctant to use psychiatric medications (Petersen et al. 2011, 2015) and reduce use by 80% during pregnancy (Hippman and Balneaves 2018). Those who continue to use psychiatric medications often report feeling guilt or worry about harming their developing child (Eakley & Lyndon 2022).

A recent umbrella meta-analysis assessed the effects of antidepressants on birth outcomes, to clarify the size of the risks (Fabiano et al. 2025). The authors reported suggestive but inconclusive risks, with very small effects: (1) PTB (1.65 [1.34–2.02]) from antidepressant use during any trimester; (2) small for gestational age with SSRI use for depression during any trimester (1.50 [1.19–1.90]); and (3) major congenital (1.24 [1.09–1.40]) or cardiac malformations (1.28 [1.11–1.47]) with first-trimester paroxetine use for depression or anxiety. While the lack of convincing evidence may be reassuring to physicians and expectant mothers, this umbrella review also highlighted limited well-conducted, controlled studies to adequately advise women of risks. Indeed, 95% of the included meta-analyses in the review were rated as either low or critically low in quality, with many of the underlying studies demonstrating biased study design, inconsistent results, indirectness, imprecision and/or publication bias.

Disentangling the effects of the underlying depression and its severity from the effect of antidepressants is challenging and can only be investigated if both are measured prospectively during pregnancy. While other large studies have investigated their independent contributions to risk, depression is often not directly assessed *during* pregnancy – presence or absence of depression is typically based on medical records of a diagnosis of depression *prior* to pregnancy, introducing recall bias (Amit et al. 2024).

The *Growing up in New Zealand* (GUiNZ) cohort provides a unique opportunity to investigate the independent influence of various risk factors, including antidepressant exposure and antenatal depression, on birth outcomes. This study collected data on depression symptoms relating to the third trimester and linked to birth records on weight and gestational age. As such, the data allow for an investigation into the independent roles of depression and antidepressant use on gestational age, birth weight, PTB (< 37-weeks gestation) and LBW (< 2500 g).

## Materials and methods

### Participants and general procedure

Participants were mothers in the GUiNZ longitudinal study, which examines six interrelated domains of child development: psychological and cognitive development, health and wellbeing, education, culture and identity, family and whānau (extended family), and neighbourhoods and societal context (Morton et al. 2015). Mothers were recruited during pregnancy from three contiguous former District Health Board (DHB) regions (Auckland, Counties-Manukau and Waikato) covering around one-third of NZ’s population, with the aim of maximising the socioeconomic and ethnic diversity of the cohort. All women with due dates between 25 April 2009 and 25 March 2010 were eligible. Ethics approval was provided by The NZ Ministry of Health Northern Y Regional Ethics Committee (NTY/08/06/055), and all participating women gave written and informed consent. For a more comprehensive account of cohort study design and sample recruitment, see Morton et al. (2013). Clinical trial number: not applicable.

The antenatal data were collected through face-to-face computer-assisted interviews occurring in participants’ homes. At 6-weeks follow-up, brief computer assisted telephone interviews were conducted, and perinatal health records were linked. Data were collected from 6,822 women at the antenatal data collection wave (DCW; 5,664 during the third trimester, 1,158 after birth) and 6,759 women at 6-weeks. Amongst the women with 6-week data, the majority (98.7%; *n* = 6668) had singleton births. In cases of multiple births, one child was randomly selected for inclusion to avoid violations to the assumption of independence. All variables described below were measured during the antenatal DCW, except for birth weight, gestational age and child’s gender, which were obtained through perinatal data linkage.

## Measures

### Antidepressant use

Women in the GUiNZ sample were asked whether they took antidepressants during the first trimester and after the first trimester. A derived variable was created to categorise antidepressant use during pregnancy. Participants were categorised as ‘yes’ if they reported taking antidepressants during either period, and ‘no’ otherwise.

## Antenatal depression

Antenatal depression symptoms were assessed using the EPDS, a 10-item self-report screening tool (Cox et al. 1987). Total scores range from 0 to 30, with higher scores indicating greater levels of distress and a score ≥ 13 indicating moderate to severe symptoms. The EPDS has been validated for use during pregnancy (Kozinszky and Dudas 2015) and is considered the most extensively validated tool for identifying antenatal depression (Sambrook Smith et al. 2022). The cut-off score of *≥* 13 has a sensitivity and specificity of 0.83 and 0.90, respectively, for major depression in pregnancy (National Collaborating Centre for Mental Health (UK) 2007).

## Unmedicated depression

A composite variable was developed to classify unmedicated depression in pregnancy, based on antidepressant use and EPDS score. Participants were grouped into three categories: Unmedicated depression (EDPS *≥* 13, no antidepressants; *n* = 5,200, 76.9% of cohort at 6-weeks), Antidepressant exposure (any use in pregnancy; *n* = 197, 2.9%) and Neither (*n* = 715, 10.6%).

### Birth outcomes

Birth weight ranged from 678 to 5850 g. Infants weighing less than 2500 g were classified as having LBW, while those weighing 2500 g or more were categorised as having average or greater birth weight.

Gestational age ranged from 25- to 46-weeks. Births before 37-weeks were classified as preterm, whereas those occurring at or beyond 37-weeks were considered full term.

## Control variables

A range of antenatal and sociodemographic variables were adjusted for in analyses. Antenatal control variables included: planned pregnancy (yes or no), parity (first-born or subsequent birth), alcohol consumption during pregnancy (alcohol or no alcohol, based on alcohol intake during and after the first trimester), and smoking during pregnancy (smoking or no smoking).

Sociodemographic variables included: mother’s education (no secondary school qualification, secondary school qualification, diploma/trade certificate, bachelor’s degree, higher degree), mother’s age during pregnancy, mother’s relationship status during pregnancy (no relationship, dating not cohabiting, cohabiting or married/civil union), mother’s self-prioritised ethnicity using Statistics New Zealand’s Level 1 categorisation (European, Māori, Pacific, Asian, Other) (Didham 2005), child’s gender (male or female), rurality (urban or rural), and area-level deprivation based on the NZDep2006 Index (low [deciles 1–3], medium [deciles 4–7], high [deciles 8–10]) (Salmond et al. 2007).

### Data analysis

Descriptive statistics were produced for the overall sample and stratified by the unmedicated depression variable. Multiple linear regression analyses were conducted to examine whether antidepressant use and antenatal depression (EDPS score) were associated with changes in birth weight and gestational age. To evaluate whether antidepressant use and antenatal depression were associated with an increased risk of more clinically significant birth outcomes (LBW and PTB), logistic regression analyses were also conducted. Control variables specified above were adjusted for in all regression models. Analyses were conducted using RStudio (version 2024.04.2).

## Results

### Descriptive results

Of the 6,759 women with 6-week data, 4.2% had a child with LBW and 5.6% had PTB (Table 1). Within this cohort, 2.9% of women were on antidepressants and 10.6% of women experienced unmedicated depression.

**Table 1:** Frequency distribution of categorical variables for the overall cohort and stratified by unmedicated depression status

		Unmedicated depression status		
	Full cohort (*N* = 6,759)	Neither (*N* = 5200)	Antidepressant exposure (*N* = 197)	Unmedicated depression (*N* = 715)
	n (%)	n (%)	n (%)	n (%)
Birth weight				
Average or above	6464 (95.6)	4991 (96.0)	~ 190^a^	671 (93.8)
Low birth weight	284 (4.2)	199 (3.8)	s	43 (6.0)
Preterm birth				
Term	6363 (94.1)	4933 (94.9)	180 (91.4)	655 (91.6)
Preterm	380 (5.6)	258 (5.0)	17 (8.6)	59 (8.3)
Planned pregnancy				
Yes	4061 (60.1)	3296 (63.4)	105 (53.3)	286 (40.0)
No	2667 (39.5)	1886 (36.3)	92 (46.7)	427 (59.7)
Alcohol during pregnancy				
No alcohol	4829 (71.4)	3734 (71.8)	123 (62.4)	489 (68.4)
Alcohol	1911 (28.3)	1459 (28.1)	73 (37.1)	226 (31.6)
Smoking during pregnancy				
No smoking	5464 (80.8)	4722 (90.8)	171 (86.8)	564 (78.9)
Smoking	634 (9.4)	461 (8.9)	25 (12.7)	148 (20.7)
Mother’s education				
No secondary school	479 (7.1)	306 (5.9)	19 (9.6)	88 (12.3)
Secondary school	1608 (23.8)	1184 (22.8)	56 (28.4)	210 (29.4)
Diploma/Trade certificate	2067 (30.8)	1538 (29.6)	60 (30.5)	267 (37.3)
Bachelor’s degree	1528 (22.6)	1260 (24.2)	35 (17.8)	94 (13.1)
Higher degree	1058 (15.7)	902 (17.3)	27 (13.7)	54 (7.6)
Mother’s relationship status				
No relationship	327 (4.8)	226 (4.3)	23 (11.7)	78 (10.9)
Dating, not cohabiting	255 (3.8)	184 (3.5)	10 (5.1)	61 (8.5)
Cohabiting	1707 (25.3)	1398 (26.9)	67 (34.0)	241 (33.7)
Married or civil union	3815 (56.4)	3380 (65.0)	97 (49.2)	332 (46.4)
Mother’s ethnicity				
European	3586 (53.1)	2899 (55.8)	157 (79.7)	245 (34.3)
Māori	939 (13.9)	679 (13.1)	20 (10.2)	140 (19.6)
Pacific	986 (14.6)	654 (12.6)	s	201 (28.1)
Asian	992 (14.7)	775 (14.9)	s	99 (13.8)
Other	237 (3.5)	184 (3.5)	s	29 (4.1)
Child’s gender				
Male	3490 (51.6)	2674 (51.4)	102 (51.8)	369 (51.6)
Female	3269 (48.4)	2526 (48.6)	95 (48.2)	346 (48.4)
Parity				
First born	2833 (41.9)	2197 (42.3)	74 (37.6)	290 (40.6)
Subsequent	3919 (58.0)	3003 (57.8)	123 (62.4)	425 (59.4)
Rurality				
Rural	464 (6.9)	378 (7.3)	20 (10.2)	42 (5.9)
Urban	6295 (93.1)	4822 (92.7)	177 (89.8)	673 (94.1)
Area-level deprivation				
Low	1684 (24.9)	1351 (26.0)	56 (28.4)	118 (16.5)
Medium	2469 (36.5)	1948 (37.5)	76 (38.6)	222 (31.0)
High	2604 (38.5)	1899 (36.5)	65 (33.0)	375 (52.4)

^a^Count rounded to the nearest 10; percentage not provided to prevent calculation of suppressed value. s = suppressed due to cell count < 10. Percentages may not add to 100% due to missing data

Descriptive statistics for the cohort overall and stratified by unmedicated depression status are presented in Table 1 for categorical variables and Table 2 for continuous data. Mothers in the Neither and Antidepressant groups had a similar proportion of children born of average or above birth weight (~ 95%). The unmedicated depression group had a higher proportion of LBW children (6.0%) compared to the Neither group (3.8%), with low counts observed for the antidepressant group. However, when examining birth weight as a continuous variable, similar mean birth weights were observed across Antidepressant (M = 3461.91, SD = 539.69) and Unmedicated depression groups (M = 3455.96, SD = 640.57), with both groups showing slightly lower birth weights than the Neither group (M = 3511.78, SD = 556.58).

**Table 2:** Descriptive statistics for continuous variables, presented for the overall cohort and stratified by unmedicated depression status

		Unmedicated depression status		
	Full cohort (*N* = 6,759)	Neither (*N* = 5200)	Antidepressant exposure (*N* = 197)	Unmedicated depression (*N* = 715)
	M (SD)	M (SD)	M (SD)	M (SD)
Birth weight	3497.27 (568.14)	3511.78 (556.58)	3461.91 (539.69)	3455.96 (640.57)
Gestational age	39.13 (1.78)	39.20 (1.71)	38.72 (1.78)	38.90 (2.12)
EPDS score	6.25 (5.03)	4.85 (3.57)	8.52 (5.59)	15.84 (2.80)
Mother’s age at birth	30.06 (5.97)	30.31 (5.85)	30.23 (5.59)	27.72 (6.20)

*EPDS* Edinburgh Postnatal Depression Scale

The Neither group had the lowest rate of PTB (5%), with approximately 8–9% of women in the Antidepressant and Unmedicated depression groups experiencing PTB. Mean gestational age was similar across all three groups (~ 39 weeks).

### Birth weight analytical analyses

Neither Antidepressant exposure nor EPDS score were associated with birth weight as a continuous variable (Table 3). However, when examining birth weight as a categorical variable, EPDS score was associated with a small increased odds of LBW (OR = 1.03, *p* <.05; Table 4).

**Table 3:** Multiple linear regression results modelling birth weight and gestational age as continuous outcome variables

	Birth weight			Gestational age		
	B (SE)	95% CI	t	B (SE)	95% CI	t
Antidepressant exposure						
No						
Yes	−57.68 (40.19)	−136.46, 21.11	−1.44	−0.40 (0.13)	−0.65, −0.15	−3.09**
EPDS score	−2.41 (1.46)	−5.27, 0.46	−1.65	−0.02 (0.005)	−0.03, −0.01	−4.58**
Planned pregnancy						
Yes						
No	6.20 (16.86)	−26.84, 39.25	0.37	−0.12 (0.05)	−0.23, −0.02	−2.26*
Alcohol during pregnancy						
No alcohol						
Alcohol	5.58 (16.29)	−26.35, 37.52	0.34	0.18 (0.05)	0.08, 0.28	3.47**
Smoking during pregnancy						
No smoking						
Smoking	−218.15 (26.23)	−269.58, −166.72	−8.32**	−0.20 (0.08)	−0.36, −0.03	−2.37*
Mother’s education						
No secondary school						
Secondary school	69.49 (32.14)	6.49, 132.50	2.16*	0.12 (0.10)	−0.08, 0.32	1.15
Diploma/Trade certificate	82.45 (31.69)	20.32, 144.57	2.60*	0.13 (0.10)	−0.07, 0.33	1.31
Bachelor’s degree	86.06 (34.66)	18.11, 154.01	2.48*	0.24 (0.11)	0.02, 0.46	2.15*
Higher degree	91.45 (36.58)	19.75, 163.16	2.50*	0.32 (0.12)	0.09, 0.55	2.71*
Mother’s age	−6.37 (1.42)	−9.15, −3.58	−4.48**	−0.03 (0.005)	−0.04, −0.03	−7.59**
Mother’s relationship status						
No relationship						
Dating, not cohabiting	−59.31 (46.44)	−150.36, 31.73	−1.28	−0.20 (0.15)	−0.49, 0.09	−1.36
Cohabiting	52.32 (33.89)	−14.12, 118.76	1.54	0.06 (0.11)	−0.15, 0.27	0.54
Married or civil union	63.77 (34.83)	−4.52, 132.05	1.83	0.01 (0.11)	−0.21, 0.23	0.08
Mother’s ethnicity						
European						
Māori	4.21 (24.13)	−43.09, 51.51	0.17	0.02 (0.08)	−0.14, 0.17	0.20
Pacific	115.62 (24.86)	66.89, 164.36	4.65**	0.06 (0.08)	−0.10, 0.21	0.73
Asian	−297.34 (22.44)	−341.33, −253.36	−13.25**	−0.21 (0.07)	−0.35, −0.07	−2.91**
Other	−92.86 (38.68)	−168.69, −17.03	−2.40*	−0.07 (0.12)	−0.31, 0.18	−0.53
Child’s gender						
Male						
Female	−100.93 (14.09)	−128.54, −73.32	−7.17**	0.05 (0.05)	−0.04, 0.14	1.15
Parity						
First born						
Subsequent	143.54 (15.48)	113.19–173.90	9.27**	−0.04 (0.05)	−0.13, 0.06	−0.71
Rurality						
Rural						
Urban	−3.91 (27.99)	−58.77, 50.96	−0.14	−0.09 (0.09)	−0.27, 0.09	−1.01
Area-level deprivation						
Low						
Medium	17.21 (18.50)	−19.05, 53.47	0.93	0.01 (0.06)	−0.10, 0.13	0.24
High	10.23 (20.92)	−30.77, 51.24	0.49	0.11 (0.07)	−0.02, 0.24	1.67

*EPDS* Edinburgh Postnatal Depression Scale. *p <.05, **p <.001

**Table 4:** Multiple logistic regression results modelling low birth weight and preterm birth as categorical outcome variables

	Low birth weight				Preterm			
	B (SE)	OR	95% CI	z	B (SE)	OR	95% CI	z
Antidepressant exposure								
No								
Yes	0.005 (0.35)	1.00	0.47, 1.90	0.01	0.37 (0.27)	1.44	0.83, 2.36	1.38
EPDS score	0.03 (0.01)	1.03	1.00, 1.05	2.23*	0.04 (0.01)	1.04	1.01, 1.06	3.25**
Planned pregnancy								
Yes								
No	−0.03 (0.16)	0.97	0.72, 1.32	−0.17	0.10 (0.14)	1.10	0.84, 1.44	0.72
Alcohol during pregnancy								
No alcohol								
Alcohol	−0.38 (0.16)	0.68	0.49, 0.93	−2.36*	−0.31 (0.14)	0.73	0.56, 0.96	−2.26*
Smoking during pregnancy								
No smoking								
Smoking	0.53 (0.22)	1.70	1.10, 2.59	2.46*	0.44 (0.19)	1.56	1.06, 2.26	2.28*
Mother’s education								
No secondary school								
Secondary school	−0.11 (0.29)	0.89	0.52, 1.60	−0.39	−0.16 (0.25)	0.85	0.53, 1.41	−0.65
Diploma/Trade certificate	−0.07 (0.28)	0.93	0.55, 1.65	−0.25	−0.21 (0.24)	0.81	0.51, 1.33	−0.86
Bachelor’s degree	−0.32 (0.31)	0.73	0.40, 1.36	−1.03	−0.26 (0.27)	0.77	0.46, 1.32	−0.96
Higher degree	−0.57 (0.34)	0.57	0.29, 1.11	−1.68	−0.55 (0.29)	0.58	0.33, 1.03	−1.89
Mother’s age	0.05 (0.01)	1.05	1.03, 1.08	3.97**	0.04 (0.01)	1.04	1.02, 1.06	3.41**
Mother’s relationship status								
No relationship								
Dating, not cohabiting	−0.03 (0.38)	0.97	0.45, 2.03	−0.08	−0.17 (0.35)	0.84	0.41, 1.66	−0.48
Cohabiting	−0.30 (0.27)	0.74	0.45, 1.28	−1.11	−0.30 (0.25)	0.74	0.46, 1.22	−1.23
Married or civil union	−0.63 (0.28)	0.53	0.31, 0.95	−2.24*	−0.29 (0.25)	0.75	0.46, 1.26	−1.13
Mother’s ethnicity								
European								
Māori	−0.18 (0.22)	0.84	0.54, 1.28	−0.80	−0.23 (0.20)	0.79	0.53, 1.16	−1.18
Pacific	−0.65 (0.27)	0.52	0.30, 0.87	−2.42*	−0.36 (0.21)	0.70	0.46, 1.05	−1.67
Asian	0.59 (0.19)	1.80	1.24, 2.59	3.12**	0.02 (0.18)	1.02	0.72, 1.44	0.12
Other	0.13 (0.34)	1.14	0.55, 2.12	0.38	−0.17 (0.32)	0.84	0.42, 1.52	−0.53
Child’s gender								
Male								
Female	0.13 (0.13)	1.14	0.88, 1.47	1.00	−0.09 (0.11)	0.91	0.73, 1.14	−0.78
Parity								
First born								
Subsequent	−0.43 (0.14)	0.65	0.49, 0.86	−3.02**	−0.19 (0.13)	0.83	0.65, 1.06	−1.51
Rurality								
Rural								
Urban	−0.55 (0.23)	0.57	0.38, 0.91	−2.46*	−0.20 (0.21)	0.82	0.55, 1.25	−0.98
Area-level deprivation								
Low								
Medium	0.05 (0.17)	1.05	0.75, 1.49	0.29	0.05 (0.15)	1.05	0.79, 1.41	0.36
High	0.17 (0.19)	1.19	0.81, 1.74	0.89	−0.12 (0.17)	0.89	0.64, 1.24	−0.69

*EPDS* Edinburgh Postnatal Depression Scale. *p <.05, **p <.001

### Gestational age and preterm birth analyses

When examining gestational age as a continuous variable, both antidepressant exposure (*b* = −0.40, *p <*.001) and greater EPDS score (*b* = −0.02, *p <*.001) were significantly associated with a slightly earlier gestational age (Table 3).

The logistic regression results examining the odds of PTB indicated that EPDS score, but not antidepressant exposure, was a significant predictor. Higher EPDS score was associated with a slightly increased odds of PTB (OR = 1.04, *p* <.001; Table 4).

## Discussion

This study investigated the independent associations between antenatal depressive symptoms, antidepressant use and two key neonatal health outcomes, birth weight and gestational age. Both outcomes were examined using continuous and categorical definitions. This approach allowed us to evaluate whether antenatal depression and antidepressant use were associated with subtle changes in birth weight and gestational age, as well as their potential influence on more clinically meaningful thresholds – namely, LBW (< 2500 g) and PTB (< 37 weeks’ gestation).

Our findings showed no association between antidepressant use and birth weight, whether treated as a continuous variable or categorised as LBW, consistent with other smaller studies (Oude Weernink et al. 2024; Wisner et al. 2013). Antenatal depressive symptoms were also not associated with birth weight as a continuous outcome, but were linked to a small increase in the odds of LBW (OR = 1.03), corresponding to an approximate 3% increase in odds per one-point increase in EPDS score. Regarding gestational age, both antidepressant use and depressive symptoms were associated with slightly earlier delivery when treated as continuous outcomes. Specifically, antidepressant exposure was associated with a 0.40-week (approximately 2.8-day) reduction in gestational age, and each one-point increase in EPDS score corresponded to a 0.02-week (around 3–4 h) reduction. However, when assessing PTB as a categorical outcome, only depressive symptoms – not antidepressant use – were significantly associated with a small increased odds (OR = 1.04), representing an approximate 4% increase in odds per EPDS point. Although statistically significant, these effect sizes were small and likely to be of limited clinical significance at the individual level. These results contrast with other studies that have demonstrated an independent association between antidepressants and PTB, even when accounting for maternal depression (Wisner et al. 2009; Yonkers et al. 2012). In general, effect sizes in these studies were small. Our small sample of women on antidepressants (*n* = 197) may have affected power to detect small effects.

Rates of preterm birth were similar among women in the antidepressant group and those with unmedicated depression (8.6% vs. 8.3%). However, interpretation of these rates is limited by potential confounding by indication, as women who use antidepressants during pregnancy may differ from those with unmedicated symptoms in illness history, symptom severity at other time points, and access to care. Accordingly, these findings should not be interpreted as conclusive evidence that antidepressant treatment either increases or decreases obstetric risk in this cohort.

From a public health perspective, even small increases in PTB and LBW may carry population-level implications. Given that untreated depression was identified as a risk factor for both conditions, investing in ways to reduce this risk factor could save the health care system millions of dollars per annual cohort. With approximately 60,000 births per year in Aotearoa New Zealand, our study allows for an estimate of around 6,300 mothers experiencing untreated depression (10.6%). With an 8.3% rate of PTB in this sub-cohort of women (3.3% higher than the rate in the nondepressed group), this could equate to about 208 extra PTBs annually based on untreated depression. Given the estimated cost of each PTB is NZ$28,000 (Newnham et al. 2022) - accounting for birth, neonatal care, delivery costs, intervention and schooling care - the total annual cohort cost that could be attributable to adverse maternal mental health in pregnancy is approximately 5.8 million. While this estimate is speculative and there may be other factors significantly contributing to this increase as well, it still highlights the importance of investing in prevention and treatment strategies that not only manage depressive symptoms effectively but also support healthy birth outcomes.

For many women, antidepressants may be the only accessible treatment option for pregnant women experiencing depression, particularly in public health systems where therapy is limited to those with very severe symptoms or private means. However, the availability of non-pharmacological treatments may be particularly important for meeting the cultural preferences of diverse communities. Studies have highlighted a strong preference for holistic and community-based approaches in some Māori, Pacific, and Asian populations, such as the use of whānau- or community-led models of care or spiritual healing practices (Agnew et al. 2024; Kishore et al. 2011; Lauber and Rössler 2007; Metcalfe et al. 2013). Emerging evidence suggests that nutritional interventions and micronutrient supplementation may benefit both maternal mood and birth outcomes (Bradley et al. 2024; Heaton et al. 2025), with birth outcomes reducing below national averages, presenting a promising area for future research. Further exploration of culturally responsive and accessible alternatives to pharmacological treatment is warranted.

### Strengths and limitations

This study’s strengths include its large, longitudinal, and socio-demographically diverse cohort, which was broadly reflective of the key demographics Aotearoa New Zealand’s pregnant population at the time (Morton et al. 2013). The availability of detailed antenatal data allowed us to control for a range of potential confounders, including maternal sociodemographic characteristics and prenatal health behaviours. The use of both continuous and categorical measures of birth weight and gestational age enables a more nuanced understanding of risk, capturing both subtle differences and clinically significant variations. By distinguishing between unmedicated depression and antidepressant use during pregnancy, our approach contributes to a growing literature that aims to isolate the effects of pharmacological treatment from the underlying condition being treated.

However, several limitations should also be acknowledged. Depression was assessed via the EPDS, a validated and widely used screening tool, but not a diagnostic instrument. In addition, while most assessments were conducted during the third trimester, a subset of women were assessed postnatally, which may introduce recall bias in reporting antenatal depressive symptoms. The timing of the assessment may also not fully capture the duration or severity of depressive symptoms across pregnancy. Antidepressant use was self-reported and not verified against pharmaceutical dispensing records, and data on antidepressant type, dosage, timing, or treatment adherence was unavailable. As a result, we were unable to examine whether associations differed by medication class, dose, or duration of exposure, which may have obscured heterogeneity in effects. Furthermore, no information was collected on psychological therapies or complementary treatments.

Additionally, while the GUiNZ sample is diverse, it may slightly overrepresent certain ethnic groups relative to the national population. However, comparisons with national data suggest that the sample remains highly representative (Svardal et al., 2021). The rate of PTB in our cohort was approximately 1.8% lower than the national average at the time (7.4%; Health New Zealand - Te Whatu Ora n.d.), which may reflect a ‘healthy volunteer’ effect or possible enhanced engagement and care associated with participation in a longitudinal study.

Finally, given the observational nature of the study, causal inferences cannot be made. In addition, the relatively small number of women exposed to antidepressants limited power to detect small effects or to examine heterogeneity in treatment history and characteristics. Future studies could therefore benefit from quasi-causal methodology using population-level administrative data or extended longitudinal cohort studies with family linkages, which may offer substantially larger sample sizes and greater capacity to address unmeasured confounding (Richmond-Rakerd et al., 2024). For example, sibling comparison designs, where siblings differ in their exposure to antidepressants or maternal depression during pregnancy, allow for the control of unmeasured familial and genetic confounding when assessing birth outcomes. Furthermore, linkage of maternity and birth records to pharmaceutical dispensing records, as is possible with linked nationwide administrative registers such as the New Zealand Integrated Data Infrastructure or Scandinavian registers (Milne et al. 2019, 2022; Richmond-Rakerd et al. 2024; Thygesen et al. 2011), would allow for more precise tracking of antidepressant exposure, include medication type, dosage and timing across pregnancy, thereby strengthening inference regarding potential treatment impacts.

## Conclusions

This study provides further evidence that both antenatal depression and antidepressant use are independently associated with slightly earlier gestational age. Depression symptoms were also associated with small increases in the odds of LBW and PTB, whereas antidepressants were not. While the effects observed were small, they have potential public health relevance. These findings reinforce the importance of accessible, evidence-based treatment options for perinatal depression, including pharmacological and non-pharmacological approaches, and support the need for ongoing investment in culturally appropriate, holistic maternal mental health care.

## Data Availability

The data analysed in this study are derived from the Growing Up in New Zealand longitudinal cohort and are subject to licensing and access restrictions. These datasets are not publicly available. Researchers interested in accessing the data can apply directly through the Growing Up in New Zealand team via their official portal: https://www.growingup.co.nz/ . Access is granted upon approval by the data custodians under applicable terms of use.
